# Risk Factors for Middle East Respiratory Syndrome Coronavirus Infection among Camel Populations, Southern Jordan, 2014–2018

**DOI:** 10.3201/eid2709.203508

**Published:** 2021-09

**Authors:** Peter Holloway, Matthew Gibson, Neeltje van Doremalen, Stephen Nash, Tanja Holloway, Michael Letko, Jacqueline M. Cardwell, Bilal Al Omari, Ahmad Al-Majali, Ehab Abu-Basha, Punam Mangtani, Vincent J. Munster, Javier Guitian

**Affiliations:** The Royal Veterinary College, Hatfield, UK (P. Holloway, J.M. Cardwell, J. Guitian);; Glasgow University, Glasgow, Scotland, UK (M. Gibson);; National Institute of Allergy and Infectious Diseases, National Institutes of Health, Hamilton, Montana, USA (N. van Doremalen, M. Letko, V.J. Munster);; London School of Hygiene and Tropical Medicine, London, UK (S. Nash, T. Holloway, P. Mangtani);; Jordan University of Science and Technology, Irbid, Jordan (B. Al Omari, A. Al-Majali, E. Abu-Basha)

**Keywords:** MERS-CoV, camels, risk factors, epidemiology, Jordan, zoonoses, Middle East respiratory syndrome coronavirus, viruses, respiratory infections

## Abstract

After the first detection of Middle East respiratory syndrome coronavirus (MERS-CoV) in camels in Jordan in 2013, we conducted 2 consecutive surveys in 2014–2015 and 2017–2018 investigating risk factors for MERS-CoV infection among camel populations in southern Jordan. Multivariate analysis to control for confounding demonstrated that borrowing of camels, particularly males, for breeding purposes was associated with increased MERS-CoV seroprevalence among receiving herds, suggesting a potential route of viral transmission between herds. Increasing age, herd size, and use of water troughs within herds were also associated with increased seroprevalence. Closed herd management practices were found to be protective. Future vaccination strategies among camel populations in Jordan could potentially prioritize breeding males, which are likely to be shared between herds. In addition, targeted management interventions with the potential to reduce transmission between herds should be considered; voluntary closed herd schemes offer a possible route to achieving disease-free herds.

Middle East respiratory syndrome (MERS) coronavirus (MERS-CoV) represents 1 of 3 major zoonotic coronaviruses to have emerged with global impact in the past 2 decades, alongside severe acute respiratory syndrome coronavirus (SARS-CoV-1) in 2002–2003 and severe acute respiratory syndrome coronavirus 2 (SARS-CoV-2) from 2019 onward (*1*). The earliest known outbreak of MERS-CoV began in a hospital in Zarqa, Jordan, in April 2012 (*2*,*3*). Since that time, >2,500 cases and 880 deaths (case-fatality rate of 34%) have been reported across 27 countries worldwide (*4*). The first detection of positive MERS-CoV by serologic testing in camels was also from Zarqa, Jordan, in 2013 (*5*); camels were later confirmed as the reservoir for MERS-CoV infection in humans (*6*) and bats the likely ancestral host (*7*).

Most confirmed MERS-CoV cases have occurred within the Arabian Peninsula; Saudi Arabia, the location of ≈80% of all human cases, is the epicenter (*8*). Phylogenetic analyses of viral sequences isolated from camels and humans suggest that multiple camel-to-human spillover events have occurred since the initial MERS outbreaks in 2012 (*9*). Although humans sometimes represent a dead-end host, secondary human-to-human infection does occur, leading in some cases to large-scale outbreaks in hospital settings, such as those seen in Saudi Arabia and South Korea in recent years (*10*,*11*). Whereas infection in camels might be subclinical or cause mild upper respiratory symptoms (*12*,*13*), infection in humans can range from asymptomatic to severe acute respiratory disease or death (*14*).

The World Health Organization has declared MERS-CoV a priority disease in its Research and Development Blueprint program as a public health risk of epidemic potential (*15*); vaccination of camels is a potential key component of future disease control strategies (*16*). Although MERS-CoV is widespread among camel populations in Africa, the Middle East, and South Asia, its epidemiology within these populations remains poorly understood, particularly with regard to viral transmission routes and risk factors for infection (*17*). Such knowledge is urgently needed if camel vaccines currently in development are to be deployed effectively (*18*–*21*) and if management interventions with the potential to contribute to disease control are to be identified. We addressed these key knowledge gaps through 2 large-scale, consecutive epidemiologic surveys among camel populations in southern Jordan, close to the border of Saudi Arabia.

## Methods

### Study Design and Study Population

**Figure 1:**
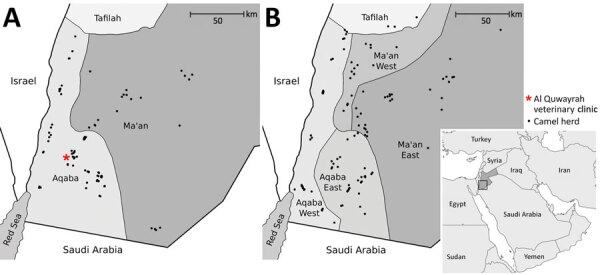
Location of camel herds sampled for Middle East respiratory syndrome coronavirus in southern Jordan, February 2014–December 2015 and October 2017–October 2018. A) 2014–2015 study; B) 2017–2018 study. Samples were taken from camels from 97 herds in the 2014–2015 study and from 121 herds in the 2017–2018 study. In the 2017–2018 study, because of local grazing movements, 3 herds selected from the Jordanian Ministry of Agriculture list for Ma’an West were sampled in the neighboring region, Tafilah, and results from these herds attributed to Ma’an West.

We conducted 2 distinct studies during February 2014–December 2015 and October 2017–October 2018. Both studies were conducted in Aqaba and Ma’an governorates of southern Jordan, an area with ≈8,000 camels (according to Jordanian Ministry of Agriculture [MoA] data) and 550 km of desert border with Saudi Arabia to the south and east (Figure 1).

In the 2014–2015 study, because of the absence of an adequate sampling frame, we conducted nonprobabilistic sampling among clients of a centrally located private veterinary practice in Al Quwayrah (Aqaba governorate). During the study period, the Al Quwayrah clinic closed (February 2015); the final 53 herds included in the study were recruited through local contacts of government veterinarians working in the study area. We collected serum samples from the onset, whereas collection of nasal swab specimens began in March 2015 and occurred in the final 53 herds only.

In the 2017–2018 study, we conducted multistage cross-sectional random sampling by using MoA-supplied lists of camel owners for Aqaba and Ma’an governorates organized by 4 local administrative areas (Aqaba East, Aqaba West, Ma’an East, and Ma’an West). We collected serum samples and nasal swab specimens from the onset.

In both studies, to encourage owner compliance, we sampled <12 camels per herd; in herds of <12, we sampled all camels, subject to accessibility and owner permissions. A structured questionnaire regarding potential risk factors for MERS-CoV infection was administered in the local dialect on paper (2014–2015 study) or on Android tablets using the application Open Data Kit (2017–2018 study) (https://getodk.org) to herd owners face-to-face at the time of sampling or by telephone after sampling. A veterinary surgeon clinically examined all camels included in the study to assess general health before sampling.

### Sample Storage and Laboratory Methods

Blood samples were collected in 8 mL serum vacutainer tubes and centrifuged at 2,000 RPM for 10 min, followed by serum collection and storage at −20°C. Nasal swab specimens were placed in viral transport medium and chilled before storage at −20°C (2014–2015 study) or −80°C (2017–2018 study). All laboratory testing of samples was performed at the Diagnostic Laboratory, Veterinary Health Centre, Jordan University of Science and Technology (Irbid, Jordan).

### ELISA

We tested serum samples in duplicate by using a MERS-CoV spike protein ELISA as previously described by van Doremalen et al. (*22*). In brief, maxisorp plates were coated overnight with S1 protein (Sino Biological, https://www.sinobiological.com) before blocking with 1% milk. MERS-CoV S1-specific antibodies were detected by using anti-llama IgG horseradish peroxidase-conjugated antibodies (Agrisera, https://www.agrisera.com) and subsequently developed with peroxidase-substrate reagent (KPL). Optical densities were measured at 405 nm and positivity at 3 times mean negative camel serum samples collected from United States–bred dromedary camels confirmed to be MERS-CoV–free. This assay does not cross-react with antibodies to bovine coronavirus, OC43, or SARS-CoV-1 (*23*).

### Viral RNA Extraction and MERS-CoV Detection

RNA was extracted from nasal swab specimens by using the QiaAmp Viral RNA kit (QIAGEN, https://www.qiagen.com) according to the manufacturer’s instructions <18 months after sample collection. Extracted RNA was used in a 1-step real-time reverse transcription PCR (rRT-PCR) UpE MERS-CoV assay performed on a QIAGEN Rotor-Gene instrument, with positivity set at a cycle threshold value of <40, on the basis of standard operating procedures as described in Corman et al. (*24*).

### Statistical Analysis

In each study, we separately calculated seroprevalence estimates weighted according to sample size relative to the estimated camel population (based on MoA data) and ran regression models for identification of risk factors. Because of the differences in sampling strategy, weighting was conducted by region for the 2014–2015 study and by subregion for the 2017–2018 study. In both studies, we excluded camels <6 months of age from analyses because of the potential influences of maternally derived immunity.

We conducted univariate analyses by using mixed-effects regression with herd as a random effect and camel serologic status considered a binary outcome. All potential risk factors were analyzed as categorical variables, with the exception of camel age and herd size, which were analyzed as continuous variables. Variables were herd level with the exception of age, sex, racing camel, and nasal discharge. For the 2017–2018 study (data were missing in the 2014–2015 study), we constructed a composite variable “closed herd,” which we defined as herds in which no borrowing, lending, purchasing, racing, or contact with local or distant herds occurred.

We considered variables with a p value of <0.2 for inclusion in the multivariate models, with the exception of any variables missing >10% of their values. We used the Pearson R coefficient and a threshold of 0.4 to compare collinearities between variables; we excluded colinear variables from the same multivariate model and tested in separate models. We conducted multivariate models by using mixed-effects regression with herd as a random effect and constructed using a backward stepwise method, removing the least significant variable at each step while p>0.1, unless the variable was considered an a priori factor (region, sex, and age) or the removal of the variable demonstrated a significant effect on the other variables (a change in log odds of >10%). We repeated model creation by using a forward stepwise method, beginning with a priori variables and adding new variables in order of significance, keeping variables if they showed significance of p<0.1 or changed the log odds of other risk factors by >10%. We performed all statistical analyses in R version 3.5.1 (https://cran.r-project.org) and generated mixed-effects models by using the glmer function of the R package lme4 version 1.1–21.

### Ethics Statement

Informed consent was obtained from all participating camel owners at the time of sampling, and institutional and national guidelines for care, use, and handling of animals were followed at all times. Studies were conducted with institutional review board approval by the Royal Veterinary College and London School of Hygiene and Tropical Medicine, National Institute of Allergy and Infectious Diseases, and Jordan University of Science and Technology and MoA.

## Results

### Study Results for 2014–2015

For 2014–2015, we included 433 camels with a median age of 6 years (interquartile range [IQR] 3–9 years) representing 97 herds (median herd size 11 [IQR 5–22]). We obtained blood samples from an average of 4.5 camels/herd and collected nasal swab specimens from 65% of included camels. The questionnaire was completed for 93 of 97 herds; we excluded 4 herds (17 camels) that lacked questionnaire data from the analysis of risk factors. A total of 21 questionnaires were completed at the time of sampling, and 72 were completed subsequently by telephone.

In total, 128 sampled camels (from 22 herds) were from Ma’an region and 305 (from 75 herds) were from Aqaba region. MoA records indicated an estimated population of 4,436 camels (317 herds) in Ma’an region and 3,314 camels (265 herds) in Aqaba region; we weighted adjusted seroprevalence accordingly. Of 433 camels sampled, 381 were seropositive for MERS-CoV, an unadjusted seroprevalence of 88.0% and adjusted seroprevalence of 86.8% (95% CI 82.8–90.3). Of these, 9 camels were <6 months of age, of which 4 were seropositive (44.4%). After we excluded these calves from the dataset, the adjusted seroprevalence was 88.0% (95% CI 84.1–91.4). No nasal swab specimens tested positive for MERS-CoV RNA on rRT-PCR.

**Figure 2:**
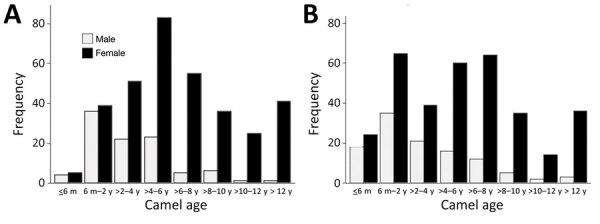
Frequency distribution of camels sampled for Middle East respiratory syndrome coronavirus in southern Jordan, February 2014–December 2015 and October 2017–October 2018, stratified by age. A) 2014–2015 study; B) 2017–2018 study.

**Figure 3:**
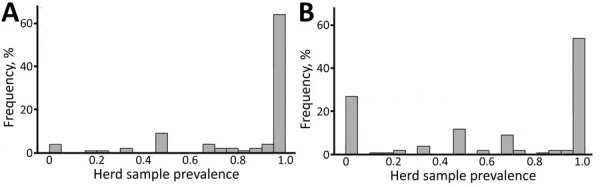
Middle East respiratory syndrome coronavirus seroprevalence among camel population in southern Jordan, stratified by age, February 2014–December 2015 and October 2017–October 2018. A) 2014–2015 study, B) 2017–2018 study. Error bars indicate 95% CIs. Numbers within gray boxes depict seropositive camels and total number of camels per age group.

Of 97 herds sampled, 93 had >1 seropositive camel (including calves <6 months of age), resulting in an unadjusted herd-level seroprevalence of 95.9% and adjusted herd-level seroprevalence of 92.3% (95% CI 83.3–97.1); median herd sample seroprevalence was 100% (IQR 80%–100%) (Figures 2, 3). Highest weight-adjusted seasonal seroprevalence was in summer (93%) and lowest was in fall (84%); winter and spring results were both 88%.

**Figure 4:**
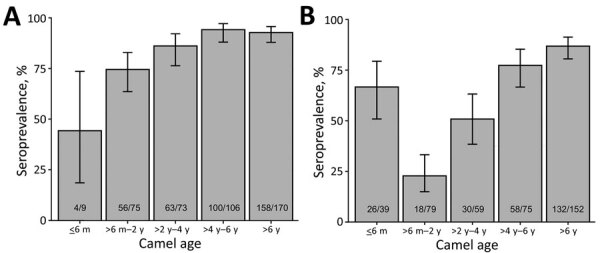
Frequency distribution of camel herd sample Middle East respiratory syndrome coronavirus seroprevalence, southern Jordan, February 2014–December 2015 and October 2017–October 2018. A) 2014–2015 study; B) 2017–2018 study

**Figure 5:**
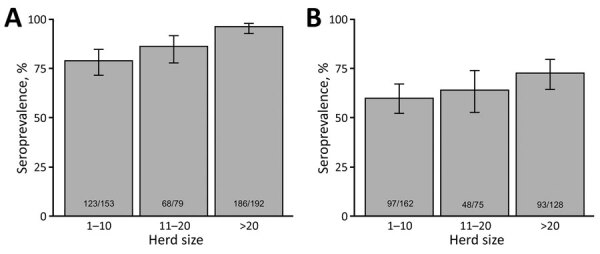
Middle East respiratory syndrome coronavirus seroprevalence among camel population in southern Jordan, stratified by herd size, February 2014–December 2015 and October 2017–October 2018. A) 2014–2015 study; B) 2017–2018 study. Error bars indicate 95% CIs. Numbers within gray boxes depict seropositive camels and total camels per herd size range.

In univariate analysis, age, sex, herd size, number of herds nearby, quarantine >3 days after purchase, borrowing of breeding males, and water source were all found to be associated with seropositivity at p<0.2, although we identified no significant correlations for these variables (Table 1, 2; Figures 4, 5). Quarantine was excluded from the multivariate models because of a high number of missing values (62%). 

**Table 1:** Descriptive statistics of Middle East respiratory syndrome coronavirus seropositivity in camel populations, Jordan, February 2014–December 2015 and October 2017–October 2018*

Variable	2014–2015 study				2017–2018 study		
Total (data missing)	No. (%) seropositive		Total (data missing)	No. (%) seropositive	
Region							
Aqaba	301	269 (89)			233	143 (61)	
Ma’an	123	108 (88)			132	95 (72)	
Subregion							
Aqaba East	NR	NR			145	93 (64)	
Aqaba West	NR	NR			88	50 (57)	
Ma’an East	NR	NR			67	55 (82)	
Ma’an West	NR	NR			65	40 (62)	
Season†							
Winter	137	122 (88)			110	56 (63)	
Spring	16	14 (88)			111	81 (75)	
Summer	185	168 (93)			NR	NR	
Fall	86	73 (84)			144	101 (70)	
Age‡							
0–6 m	9 (6)	4 (44)			39	26 (67)	
>6 m–2 y	75	56 (75)			79	18 (23)	
2 y–4 y	73	63 (86)			59	30 (51)	
4 y–6 y	106	100 (94)			75	58 (77)	
>6 y	170	158 (93)			152	132 (87)	
Sex‡							
M	94	79 (84)			84	40 (48)	
F	330	298 (90)			281	198 (70)	
Breeding male§	52 (6)	47 (90)			21 (12)	14 (67)	
Herd size							
1–10	153	123 (80)			162	97 (60)	
11–20	79	68 (86)			75	48 (64)	
>20	192	186 (97)			128	93 (73)	
No. camel herds within a 15-min drive							
<20	192 (25)	166 (86)			259	161 (62)	
>20	207	192 (93)			106	77 (73)	
Herd kept together as single group throughout the year							
No	70 (17)	64 (91)			167	98 (59)	
Yes	337	302 (90)			198	140 (71)	
Herd has contact with other local herds							
No	101 (114)	88 (87)			134	72 (54)	
Yes	209	188 (90)			231	166 (72)	
Herd has contact with distant herds							
No	265 (20)	242 (91)			176	104 (59)	
Yes	139	121 (87)			189	134 (71)	
New camels are purchased¶							
No	245 (17)	221 (90)			240	157 (65)	
Yes	162	145 (90)			125	81 (65)	
Quarantine >3 d after purchase before joining herd							
No	97 (262)	92 (95)			101	67 (66)	
Yes	65	53 (82)			24	14 (58)	
Camels borrowed for breeding purposes#							
No	164(17)	139 (85)			210	123 (59)	
Yes	243	227 (93)			155	115 (74)	
Herd-level borrowing of males							
No	164(17)	139 (85)			NR	NR	
Yes	243	227 (93)			NR	NR	
Herd-level borrowing of females							
No	322(17)	286 (89)			NR	NR	
Yes	85	80 (94)			NR	NR	
Camels loaned for breeding purposes							
No	NR	NR			203	123 (61)	
Yes	NR	NR			162	115 (71)	
Camels in the herd used for racing							
No	357 (17)	322 (90)			213	140 (66)	
Yes	50	44 (88)			152	98 (64)	
Camel is a racing camel‡							
No	NR	NR			317	214 (68)	
Yes	NR	NR			48	24 (50)	
Water source**							
Open ad lib	24 (17)	19 (79)			57	27 (47)	
Household only	19	16 (84)			100	67 (67)	
Trough only	364	331 (91)			208	144 (69)	
Spring							
No	384 (17)	348 (91)			312	214 (69)	
Yes	23	18 (78)			53	24 (45)	
Irrigation reservoir							
No	398 (17)	360 (90)			335	223 (67)	
Yes	9	6 (67)			30	15 (50)	
Tanker							
No	287 (17)	255 (89)			247	168 (68)	
Yes	120	111 (93)			118	70 (59)	
Tap							
No	111 (17)	101 (91)			198	124 (63)	
Yes	296	265 (90)			167	114 (68)	
Well							
No	356 (17)	321 (90)			276	189 (69)	
Yes	51	45 (88)			89	49 (55)	
Water source not shared with herd, household use only							
No	388	350 (90)			265	171 (65)	
Yes	19	16 (84)			100	67 (67)	
Camel has nasal discharge at time of sampling‡							
No	NR	NR			303 (54)	204 (67)	
Yes	NR	NR			8	8 (100)	
Closed herd††							
No	NR	NR			328	227 (69)	
Yes	NR	NR			37	11 (30)	

*Variables reference the 1-year period before sampling, with the exception of herd size, camel is a racing camel, and a priori variables: age, sex, and region. Because of the potential influence of maternal immunity, camels <6 m of age have been excluded from all variables except age. NR, not recorded. †Weighting adjusted. ‡Individual camel–level variables (all other variables being herd-level). §Nonracing adult males (>2 y), likely in the herd for breeding purposes. ¶Camels purchased are locally bred; Jordanian Ministry of Agriculture Camel Import Regulations and Conditions allow import only for live camels for direct slaughter. ^#^In the 2014–2015 study, results for camels are borrowed for breeding purposes (male, female, or both) and camels are borrowed for breeding purposes (male) were the same (i.e., all herds that borrowed camels for breeding purposes borrowed males, and some of these herds also borrowed females. In the 2017–2018 study, the sex of camels borrowed or lent for breeding purposes was not recorded. **Open ad lib indicates irrigation reservoir or spring water sources were used; household only indicates water source was not shared between household and herd; trough only indicates only tanker, tap, or well sources were used. ††Closed herd indicates herd owners answered no to all of the following variables: borrowing, lending, purchasing, racing, and contact with local or distant herds (2017–2018 study only, missing data 2014–2015).

**Table 2:** Univariate associations between potential risk factors and Middle East respiratory syndrome coronavirus seropositivity in camel populations, Jordan, February 2014–December 2015 and October 2017–October 2018*

Variable	2014–2015 study				2017–2018 study					
OR (95% CI)		p value	OR (95% CI)					p value
Region										
Aqaba	Referent		0.53		Referent					0.01
Ma’an	0.68 (0.18–2.30)				3.95 (1.42–12.85)					
Age, per y†	1.22 (1.08–1.39)		<0.01		1.63 (1.39–2.01)					<0.01
Sex†										
F	2.48 (0.90–6.70)		0.07		3.02 (1.28–7.09)					0.01
Herd size										
Per individual no. camels	1.05 (1.01–1.09)		0.01		1.02 (1.00–1.04)					0.08
No. camel herds within a 15 min drive										
>20	2.42 (0.72–9.07)		0.16		2.24 (0.70–7.86)					0.18
Herd kept together as single group throughout the year	0.84 (0.13–5.04)		0.85		2.88 (1.04–8.93)					0.05
Herd has contact with other local herds	1.43 (0.30–6.64)		0.63		2.97 (1.07–9.17)					0.04
Herd has contact with distant herds	0.55 (0.13–2.00)		0.36		1.86 (0.67–5.34)					0.23
New camels are purchased‡	0.77 (0.19–2.94)		0.70		1.51 (0.53–4.71)					0.44
Quarantine >3 d after purchase before joining herd	0.23 (0.03–1.55)		0.10		0.42 (0.02–6.88)					0.52
Camels borrowed for breeding purposes§	2.96 (0.87–11.42)		0.08		3.94 (1.45–12.32)					0.01
Herd-level borrowing of males	2.96 (0.87–11.42)		0.08		NR					NR
Herd-level borrowing of females	2.45 (0.49–16.62)		0.30		NR					NR
Camels loaned for breeding	NR		NR		3.28 (1.19–10.44)					0.03
Camels in herd are used for racing	0.49 (0.07–3.26)		0.44		0.89 (0.29–2.66)					0.83
Camel is a racing camel†	NR		NR		0.37 (0.09–1.44)					0.15
Water source¶										
Open ad lib	Referent		0.13		Referent					0.15
Household only	1.89 (0.05–72.38)				2.81 (0.59–14.25)					
Trough only	7.15 (0.95–70.49)				4.07 (1.01–18.88)					
Spring	0.13 (0.01–0.98)		0.05		0.20 (0.04–0.80)					0.03
Irrigation reservoir	0.05 (0.00–0.91)		0.05		0.36 (0.06–2.22)					0.27
Tanker	1.24 (0.31–5.11)		0.75		0.77 (0.25–2.37)					0.64
Tap	0.82 (0.20–3.94)		0.78		0.99 (0.35–2.86)					0.99
Well	0.57 (0.08–3.57)		0.54		0.44 (0.13–1.41)					0.16
Water source not shared with herd, household use only	0.30 (0.01–7.54)		0.45		0.96 (0.29–3.00)					0.94
Closed herd#	NR		NR		0.09 (0.01–0.39)					<0.01

*Variables reference the 1-year period before sampling, with the exception of herd size, camel is a racing camel, and a priori variables: age, sex, and region. Because of the potential influence of maternal immunity, camels <6 m of age have been excluded from all variables except age. NR, not recorded. †Individual camel–level variables (all other variables being herd-level). ‡Camels purchased are locally bred; Jordanian Ministry of Agriculture Camel Import Regulations and Conditions allow import only for live camels for direct slaughter. §In the 2014–15 study, results for camels are borrowed for breeding purposes (male and/or female) and camels are borrowed for breeding purposes (male) were the same (i.e., all herds that borrowed camels for breeding borrowed males, and some of these herds also borrowed females). In the 2017–18 study, the sex of camels borrowed or loaned for breeding was not recorded. ¶Open ad lib indicates irrigation reservoir or spring water sources were used; household only indicates water source was not shared between household and herd; trough only indicates only tanker, tap, or well sources were used. #Closed herd indicates herd owners answered no to all of the following variables: borrowing, lending, purchasing, racing, and contact with local or distant herds (2017–2018 study only, missing data 2014–2015).

Variables in the final multivariate model results were age, herd size, borrowing of males for breeding purposes, and water source (Table 3). We noted evidence of an association between camel seropositivity and borrowing of males for breeding purposes (adjusted OR [aOR] 4.18 (95% CI 1.45–12.09); p = 0.01), age per year (aOR 1.24 [95% CI 1.08–1.42]; p<0.01), and herd size per additional camel (aOR 1.04 [95% CI 1.01–1.08]; p = 0.02).

**Table 3:** Multivariate associations between potential risk factors and Middle East respiratory syndrome coronavirus seropositivity in camel populations, southern Jordan, February 2014–December 2015*

Variable	A priori adjusted OR (95% CI)†	p value	Fully adjusted OR (95% CI)‡	p value	
Age, per y§	1.21 (1.07—1.40)	<0.01	1.24 (1.08—1.42)	<0.01	
Male camels borrowed for breeding purposes	3.44 (1.09—12.25)	0.04	4.18 (1.45—12.09)	0.01	
Herd size					
Increasing individual camel nos.	1.05 (1.01—1.09)	<0.01	1.04 (1.01—1.08)	0.02	
Water source¶					
Open ad lib	Referent	0.19	Referent	0.08	
Household only	0.52 (0.01—21.39)		0.90 (0.05—16.46)		
Trough only	4.02 (0.51—40.84)		4.74 (0.93—24.08)		
Region					
Ma’an	0.56 (0.16—1.79)	0.33	0.37 (0.12—1.14)	0.08	
Sex§					
F	1.35 (0.45—3.86)	0.58	1.12 (0.38—3.26)	0.84	
Number of camel herds within a 15-min drive					
>20	2.24 (0.68—7.99)	0.18	–	–	

*Variables reference the 1-year period before sampling, with the exception of herd size, camel is a racing camel, and a priori variables: age, sex, and region. Because of the potential influence of maternal immunity, camels <6 m of age have been excluded. OR, odds ratio. †Adjusted for a priori variables: age, sex, and region. ‡2014–2015 study was adjusted for a priori variables and number of camels nearby (within a 15 min drive). §Individual camel–level variables (all other variables being herd-level). ¶Open ad lib indicates irrigation reservoir or spring water sources were used; household only indicates water source was not shared between household and herd; trough only indicates only tanker, tap, or well sources were used.

### Study Results for 2017–2018 

Blood samples and nasal swab specimens were collected from 404 camels (median age 5 years [IQR 3–8 years]) in 121 herds; an average of 3.3 camels were sampled per herd (median herd size 9 [IQR 4–17]). The questionnaire was administered to all 121 herd owners; 114 questionnaires were completed at the time of sampling, and 7 were completed subsequently by telephone. In total, 90 camels (29 herds) were sampled from Ma’an East, 70 (21 herds) Ma’an West, 152 camels (36 herds) from Aqaba East, and 92 (35 herds) from Aqaba West. MoA records described an estimated 1,909 camels (138 herds) in Aqaba East, 1,405 camels (127 herds) in Aqaba West, 3,563 camels (198 herds) in Ma’an East, and 873 camels (119 herds) in Ma’an West; we weighted adjusted seroprevalence accordingly.

Of 404 camels sampled, 264 were seropositive for MERS-CoV, for an unadjusted seroprevalence of 65.3% and an adjusted seroprevalence of 70.2% (95% CI 65.6–74.7). Of these, 26 of 39 camels <6 months of age were seropositive (66.7%), which compares with 18 (22.8%) of 79 among camels >6 months–2 years of age (OR 20.8 [95% CI 4.8–226.3]; p<0.01). After removal of calves <6 months from the dataset, the adjusted seroprevalence was 70.2% (95% CI 65.0–75.2) among 119 herds.

Of 119 herds sampled, 92 had >1 seropositive camel (including calves <6 months of age), resulting in an unadjusted herd-level seroprevalence of 77.3% and adjusted herd-level seroprevalence of 77.0% (95% CI 69.8–83.0); median herd sample seroprevalence was 75% (IQR 25%–100%) (Figures 2, 3). The highest weight-adjusted seasonal seroprevalence was in spring (75%) and the lowest in winter (63%); seroprevalence in fall was 70% (because of logistical constraints, no samples were collected during the summer).

No nasal swab specimens tested positive for MERS-CoV RNA on rRT-PCR. Nasal discharge was noted in 8 camels (2.6% [95% CI 1.4%–4.8%]) at the time of sampling (ages 3, 5, 6, 7, 7, 12, 14 and 15 years).

In the univariate analysis, the following 12 variables were found to be associated with seropositivity at p<0.2: region, age, sex, herd size, number of herds nearby, herd being kept as a single group throughout the year, contact with local herds, borrowing of camels for breeding purposes, lending of camels for breeding purposes, use of camels for racing, water source, and closed herd status (Tables 1, 2; Figures 4, 5). We identified correlations between contact with local herds and lending for breeding purposes (Pearson R coefficient = 0.46) and between borrowing for breeding purposes and lending for breeding purposes (Pearson R coefficient = 0.46).

Variables in the final multivariate model results were region, sex, age, herd size, borrowing for breeding purposes, water source, and closed herd status (Table 4). Evidence of an association was noted between camel MERS-CoV seropositivity and drinking from water trough sources only, as compared with open ad lib sources (aOR 9.48 [95% CI 1.54–58.24]; p = 0.05); borrowing camels for breeding purposes (aOR 5.07 [95% CI 1.37–18.75]; p = 0.02); location in Ma’an region (aOR 3.83 [95% CI 1.01–14.51]; p = 0.05); and increasing age per year (aOR 1.60 [95% CI 1.34–1.92]; p<0.01). We investigated the variable of lending camels for breeding purposes in a separate model, in place of camels being borrowed for breeding purposes, but did not find evidence of a significant association with seropositivity. The composite variable closed herd demonstrated evidence of a protective association with MERS-CoV seropositivity (aOR 0.08 [95% CI 0.01–0.55]; p = 0.02) when included in a separate model adjusted for the same confounders, although excluding constituent variables for closed herd, borrowing, and lending for breeding purposes.

**Table 4:** Multivariate associations between potential risk factors and Middle East respiratory syndrome coronavirus seropositivity in camel populations, southern Jordan, October 2017–October 2018

Variable*	A priori adjusted OR (95% CI)†	p value	Fully adjusted OR (95% CI)‡	p value
Age, per y§	1.60 (1.35—1.99)	<0.01	1.60 (1.34—1.92)	<0.01
Camels borrowed for breeding purposes	4.46 (1.29—21.68)	0.03	5.07 (1.37—18.75)	0.02
Water source¶				
Open ad lib	Referent	0.07	Referent	0.05
Household only	3.17 (0.44—25.78)		3.33 (0.51—21.71)	
Trough only	7.93 (1.41—65.04)		9.48 (1.54—58.24)	
Region				
Ma’an	3.28 (0.92—14.94)	0.08	3.83 (1.01—14.51)	0.05
Herd size				
Increasing individual camel nos.	1.02 (1.00—1.05)	0.05	1.00 (1.00—1.05)	0.10
Sex§				
F	1.70 (0.59—4.88)	0.32	1.38 (0.48—3.97)	0.54
No. camel herds within a 15-min drive				
>20	3.33 (0.77—17.53)	0.11	2.40 (0.53—10.84)	0.25
Herd is kept together as single group throughout the year	2.24 (0.61. 9.85)	0.23	–	–
Herd has contact with other local herds	2.34 (0.65—9.85)	0.19	–	–
Camel is a racing camel§	0.73 (0.13—4.33)	0.72	–	–
Camels are lent for breeding purposes	2.39 (0.66—10.70)	0.19	–	–
Closed herd#	0.07 (0.01—0.43)	0.01	0.08 (0.01—0.55)	0.02

*Variables reference the 1-year period before sampling, with the exception of herd size, camel is a racing camel, and a priori variables: age, sex, and region. Because of the potential influence of maternal immunity, camels <6 m of age have been excluded. †Adjusted for a priori variables: age, sex, and region. ‡2017–2018 study was adjusted for a priori variables and number of camels nearby (within a 15 min drive), herd is kept as a single group throughout the year, herd has contact with other local herds, and camel is a racing camel. §Individual camel–level variables (all other variables being herd-level). ¶Open ad lib indicates irrigation reservoir or spring water sources were used; household only indicates water source was not shared between household and herd; trough only indicates only tanker, tap, or well sources were used. #Closed herd indicates herd owners answered no to all of the following variables: borrowing, lending, purchasing, racing, and contact with local or distant herds (2017–2018 study only, missing data 2014–2015). Because of collinearity with constituent variables, the variable closed herd was included in a separate multivariate model from camels are borrowed for breeding purposes and camels are lent for breeding purposes. In this model, all variables listed continued to demonstrate significant association (p<0.05) with Middle East respiratory syndrome coronavirus seropositivity, with the exception of water source (p = 0.07).

## Discussion

Previous studies have described MERS-CoV seroprevalence among camel populations worldwide; however, substantial knowledge gaps remain, in particular with regard to factors associated with higher risk for infection, which might provide insights into viral transmission routes between and within camel herds (*16*,*17*). Such knowledge is essential if effective disease control strategies, such as targeted vaccination programs and camel management interventions, are to be appropriately designed and implemented.

Our findings suggest that borrowing male camels for breeding might serve as a transmission route for MERS-CoV between infected and uninfected camel herds in Jordan. Both studies demonstrated that borrowing camels for breeding was associated with an increase in MERS-CoV seropositivity in receiving herds. In addition, the 2014–2015 study demonstrated that the borrowing of breeding males was a significant risk, whereas the borrowing of breeding females was not (we did not record sex of camels borrowed for breeding purposes in 2017–2018).

In Jordan, as in other countries in the region, many herd owners do not own a breeding male camel because of cost or ease of management; instead, they borrow stud bulls from neighboring herds or send breeding females to herds that have a bull. These practices serve to provide spatial connectivity between infected and uninfected herds; this effect is potentially compounded by the immunosuppressive stresses of transport and joining a new herd and by the effects of male rutting behavior, in which oronasal secretions are sprayed over, or close to, breeding females (*25*,*26*).

Given evidence for the potential risk posed by borrowing breeding males, vaccination of male camels shared between herds for breeding could be prioritized when effective camel vaccines become available (*18*), particularly among small-scale extensively managed herds, such as those in Jordan. In addition, despite the challenges of artificial insemination in camelids, the introduction of an affordable artificial insemination service, where feasible, could mitigate the transmission of MERS-CoV between infected and uninfected herds (*27*). Other potential control measures could be introducing rRT-PCR testing schemes using nasal swab samples before movement between herds and quarantining of positive animals (*28*). In view of the current understanding that MERS-CoV transmission in camels occurs primarily through upper respiratory droplet, evidence for possible sexual transmission remains inconclusive, and further research is required (*12*,*29*).

Closed herd management practices were found to be significantly protective, offering a potentially valuable tool in controlling of MERS-CoV among camels; voluntary closed herd schemes are a possible route to achieving disease-free herds (*30*). Where such practices would be impractical, our findings suggest that quarantining animals before introduction to the herd offers a protective effect. On the basis of current evidence of viral shedding patterns in camels, quarantine periods of >2 weeks should be employed.

Increasing herd size was found to be associated with increased MERS-CoV seroprevalence; larger herds are thought to provide a greater host reservoir capable of sustaining viral transmission between infected and uninfected animals (*16*,*17*). In addition, the use of water troughs within herds, as opposed to open ad lib water sources, was associated with increased herd seroprevalence (although only in the 2017–2018 multivariate model when the variable borrowing for breeding was included). Although crowded troughs might be a potential route of viral transmission within herds, further research is required (*31*).

As described in other studies, seroprevalence increased significantly with age in both studies, likely associated with the increased probability of disease exposure over time and boosting of antibody levels by repeat infections (*16*,*17*). Results of the 2017–2018 study strongly suggest the presence of maternally derived immunity among calves <6 months of age, which could have relevance for future vaccination strategies (*18*). This association was less evident in the 2014–2015 study; however, only 9 camels <6 months of age were sampled in 2014–2015, compared with 39 in 2017–2018. Associations between sex and seropositive status have been previously described, but no significant associations were identified in either study (*16*,*17*,*32*).

The difference in adjusted seroprevalence observed between studies (together with differences in regional associations with seropositivity) might be explained by several factors. The factors include differences in sampling strategy (nonprobabilistic vs. probabilistic), an absence of sample collection during the 2017–2018 summer period (with seroprevalence highest in summer 2014–2015), and a possibly limited introduction of new MERS-CoV variants into the population between the study periods, with geographic spread over time (*33*). Importing of foreign camels into Jordan is strictly regulated by MoA and permitted only for animals going directly to slaughter (*34*).

The first limitation of this study is that no nasal swab specimens tested positive for MERS-CoV RNA on rRT-PCR; evidence for potential viral transmission routes were therefore suggestive instead of definitive. Possible explanations include the narrow window of nasal shedding reported in camels (<2 weeks) (*12*) and the low prevalence of nasal discharge observed, potentially reflecting a limited genetic diversity of MERS-CoV variants circulating among camels in Jordan with rapid seroconversion and clearance (*35*). Second, limited sample size resulted in considerable uncertainty on strength of associations. Third, data at the level of individual camels, particularly regarding history of movement for purchase and breeding, were limited. Such data could have supported herd-level findings and identified camels potentially infected outside the herd (depending on duration of detectable antibodies) (*36*). Fourth, in detecting an association between seropositivity and potential risk factors, assumptions were made regarding persistence of detectable antibodies (>1 year), meaning that estimates of association are potentially conservative (*37*,*38*).

In conclusion, borrowing male camels for breeding and closed herd management practices were associated with MERS-CoV infection prevalence among camel populations in Jordan, suggesting possible useful interventions to reduce transmission. In addition, older age, larger herd size, and use of water troughs within herds were also associated with seropositivity. In view of this finding, future MERS-CoV vaccination strategies among camel populations in Jordan could potentially prioritize breeding males, which are likely to be shared between herds for breeding purposes. In addition, several targeted management interventions should be considered: measures to reduce the number of camels, particularly males, shared between herds for breeding purposes (including, if feasible, introducing an affordable camel artificial insemination service at a regional or national level); maintaining a closed herd where possible, including the potential for voluntary closed herd management schemes; and quarantine practices of >2 weeks before introducing new animals to the herd. The implementation of such interventions among herds in Jordan and the wider region, alongside targeted vaccination, could reduce the prevalence of MERS-CoV among camel populations and confer a vitally protective effect on human populations associated with these herds (*39*).
